# Enantiomer-specific analysis of multi-component mixtures by correlated electron imaging–ion mass spectrometry

**DOI:** 10.1038/ncomms8511

**Published:** 2015-06-24

**Authors:** Mohammad M Rafiee Fanood, N. Bhargava Ram, C. Stefan Lehmann, Ivan Powis, Maurice H. M. Janssen

**Affiliations:** 1 LaserLaB Amsterdam and Department of Physics and Astronomy, VU University Amsterdam, De Boelelaan 1081, Amsterdam 1081 HV, The Netherlands; 2 School of Chemistry, University Park, University of Nottingham, Nottingham NG7 2RD, UK

## Abstract

Simultaneous, enantiomer-specific identification of chiral molecules in multi-component mixtures is extremely challenging. Many established techniques for single-component analysis fail to provide selectivity in multi-component mixtures and lack sensitivity for dilute samples. Here we show how enantiomers may be differentiated by mass-selected photoelectron circular dichroism using an electron–ion coincidence imaging spectrometer. As proof of concept, vapours containing ∼1% of two chiral monoterpene molecules, limonene and camphor, are irradiated by a circularly polarized femtosecond laser, resulting in multiphoton near-threshold ionization with little molecular fragmentation. Large chiral asymmetries (2–4%) are observed in the mass-tagged photoelectron angular distributions. These asymmetries switch sign according to the handedness (R- or S-) of the enantiomer in the mixture and scale with enantiomeric excess of a component. The results demonstrate that mass spectrometric identification of mixtures of chiral molecules and quantitative determination of enantiomeric excess can be achieved in a table-top instrument.

## Abstract (web summary)

Detecting enantiomers in dilute mixtures of volatile organic compounds is a challenge. Here, the authors demonstrate a method to identify enantiomers and enantiomeric excess in a multi-component mixture containing two chiral species using laser mass spectrometry and photoelectron circular dichroism.

## Graphical abstract

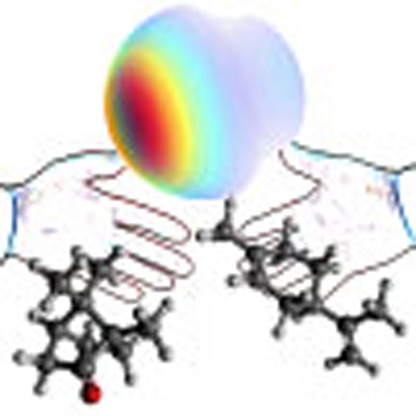

There is an increasing awareness of the benefits stemming from enantiomer-specific analysis of mixtures of volatile organics (VOCs). These advantages have long been apparent in the analysis of food odours where hundreds of chiral odorant pairs having different perceived smells are known^1^. Recent work has highlighted the role for enantiomer-specific detection of, for example, the monoterpene limonene in analysing biogenic emissions of VOCs^2^,^3^. In a related manner, detection of enantiomeric metabolites in clinical breath analysis may play an increasing role in the understanding and development of chiral pharmaceuticals. Much progress has been made in the analysis of mixtures of VOCs in such areas using direct injection mass spectrometry^4^,^5^, where ‘soft' ionization techniques can obviate the need for prior chromatographic separation, a particular advantage for field and *in vivo* studies. Yet mass spectrometry, traditionally a ‘chirally blind' technique, does not directly address the identification of enantiomers in these mixtures, providing a stimulus for the development of enhanced approaches that can provide such capabilities.

Mass spectrometric techniques for determination of single-component enantiomeric excess (e.e.) relevant to pharmaceutical development have, however, developed greatly over the past decade^6^,^7^ and rely on interactions with a known chiral reference reagent to discriminate enantiomers. These reference compounds have to be carefully chosen in advance according to the specific target species, and have themselves to be sourced in enantiopure form. Likely interference from contaminants in the analysed sample stream has also to be considered, although hybrid tandem mass spectrometric techniques can be used to reduce such interferences.

As an alternative to chemical discriminants, a fully generic chiral reference is provided by circularly polarized light (CPL), readily prepared and characterized by well-established optical techniques. Chiroptical methods, such as electronic circular dichroism and vibrational optical activity (VOA) and vibrational circular dichroism (VCD) rely on typically rather weak chiral asymmetries (0.001–0.01%) in light–matter interaction, but are widely used techniques for the detection and structural determination of solvated chiral molecules^8^,^9^.

Recently, circular dichroism in ion yields generated by CPL multiphoton laser ionization of chiral molecules has been shown to be capable of discriminating enantiomers in solvent free conditions^10^,^11^. An alternative, gas phase approach for the analysis of chiral isomer mixtures, based on phase-sensitive microwave spectroscopy, has also been demonstrated^12^,^13^. By eliminating the often indeterminate contributions from solvation^14^, gas phase studies of biomolecules can be of considerable value for developing fundamental understanding^15^ and permit the weak interactions responsible for chiral molecular recognition to be directly examined^16^,^17^.

In this report we describe a proof of concept demonstration of a direct, generic method for the simultaneous determination of enantiomeric components of a gaseous mixture by laser-based mass spectrometric detection, coupled with coincident electron imaging detection in a single table-top instrument. The detection of mass-selected parent ions in coincidence with energy dispersed electrons allows the electron spectra to be tagged by the precursor neutral. Recent work with synchrotron radiation has demonstrated the analytical potential of recording correlated ion and photoelectron ion spectra in this fashion^18^. Here, we add the convenience and additional capabilities of a laboratory-based ultrafast laser ionization source for optimized parent ion production with, crucially, the use of circular polarizations and energy- and angle-resolved imaging detection of the coincident electron. The latter provides the capability of enantiomeric differentiation by photoelectron circular dichroism (PECD)^19^,^20^,^21^. PECD derives from chiral asymmetries in the photoelectron angular distribution that, for single vacuum ultraviolet (VUV) photon ionization, routinely fall in the range 1–10%. These expectations are maintained in the recent emergence of multiphoton PECD where, again, asymmetries of up to 10% have been reported from single pure enantiomers^22^,^23^,^24^,^25^,^26^,^27^. Such large asymmetries provide more than sufficient sensitivity to identify enantiomers of even minor (that is, few %) components in a mass spectrum^28^. Hence, electron–ion coincidence imaging^29^ is here shown to create the capability to record fully multiplexed mass-tagged photoelectron circular dichroism spectra of chiral molecules in multi-component mixtures.

## Results

### Coincidence detection of electrons and ions

**Figure 1:**
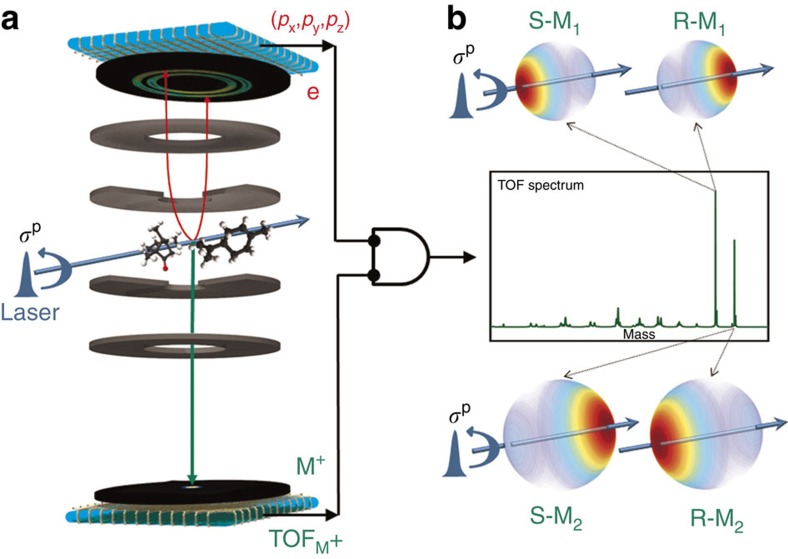
Concept and implementation of MS-PECD. (**a**) Following an ionizing laser pulse that crosses a molecular sample beam, ions and electrons are extracted in opposing directions by electric fields to reach their respective detectors. Electrons are focussed using a velocity map imaging arrangement of electrodes to yield the complete (*p*_x_,*p*_y_,*p*_z_) momentum from the time- and position-sensitive imaging detector^33^. For the present experiment mass analysis is achieved from measuring just the ion ToF. Detection of a mass-selected ion in delayed coincidence with an electron identifies particles from a single molecular ionization event and these electron–ion correlations can be used to ‘tag' the accumulated electron data by their respective parent molecular masses. Panel (**b**) shows how the mass-tagged electron angular distributions recorded with circular polarized light display a forward–backward asymmetry that can distinguish between enantiomers, while the radius maps the electron kinetic energy.

Key features of the mass-selective photoelectron circular dichroism (MS-PECD) experiment are provided in Fig. 1. Briefly the electron and ion from an individual molecular ionization by a laser pulse are projected to respective detectors by electric fields. The three-dimensional momentum distribution of the electron is recovered from its position and time-of-flight (ToF) at the detector, while the mass of the ion can be determined from its ToF. Coincident detection of the pair establishes their common origin, and allows the electron data to be ‘tagged' according to the parent molecular mass. The accumulated electron data from many such tagged coincidence detections can be used to create the species-specific photoelectron energy and angular distributions required for PECD analysis.

### Mixture analysis

**Figure 2:**
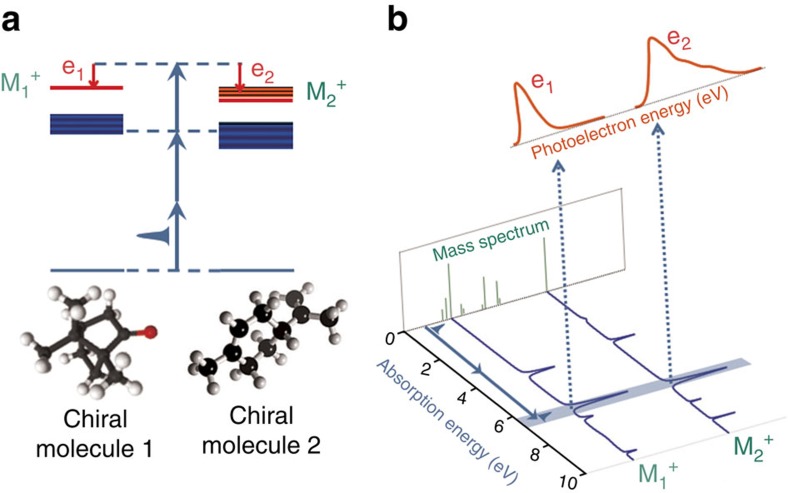
Multiphoton ionization by femtosecond laser excitation for a two component mixture. Panel (**a**) shows schematically the ‘soft', two photon-resonant, three-photon-ionization producing parent molecular ions and coincident electrons (e_1_,M_1_^+^) and (e_2_,M_2_^+^). Panel (**b**) indicates how the laser may be tuned to excite via specific resonant neutral states. The different photoelectron energy distributions (spectra) obtained from the mass-tagged coincident electrons e_1_ and e_2_ are shown schematically and may be used to help chemical identification.

As a demonstration we sample prepared mixtures containing <1% of two enantiomeric monoterpenes, camphor (C_10_H_16_O) and limonene (C_10_H_16_), in an inert carrier gas (Ne). Both terpenes occur as biogenic VOCs with stereoiosomerism of limonene, in particular, being of significant interest in odour analysis and monitoring terrestrial plant emissions^2^. Very recently, PECD asymmetries of ∼8% have been demonstrated for pure camphor enantiomers in multiphoton ionization using femtosecond (fs) laser pulses around 400 nm^21^,^22^,^23^,^24^, while multiphoton PECD asymmetries of ∼4% have been reported for limonene using fs laser pulses centred at 420 nm^27^. Fig. 2 shows schematically the simultaneously excited two-photon-resonant, three-photon ionization scheme for this mixture.

**Figure 3:**
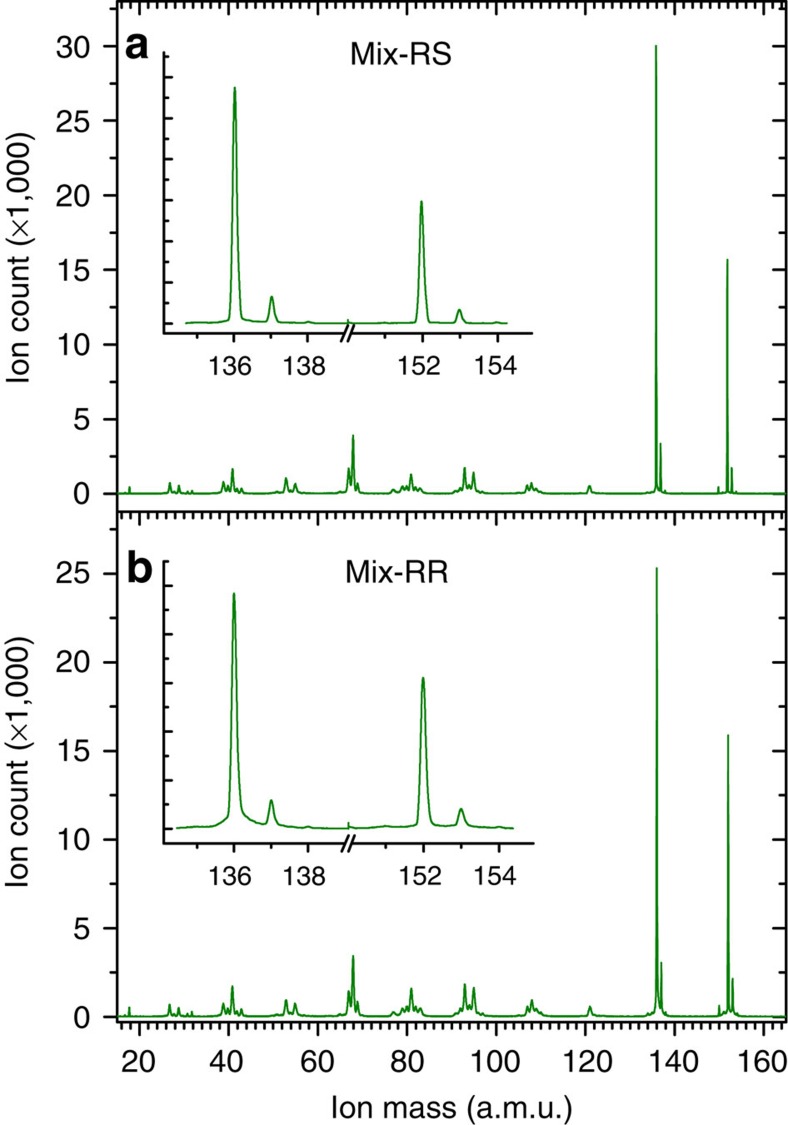
Measured time-of-flight spectra of two mixtures of limonene (136 a.m.u.) and camphor (152 a.m.u.). In panel (**a**) the ToF spectrum of a mixture containing R-limonene and S-camphor is shown, while in panel (**b**) the ToF spectrum of a mixture containing R-limonene and R-camphor is shown. The mixtures were seeded in neon and expanded through a nozzle as a molecular beam. The parent mass region is expanded in the insets. The small peaks at masses 137 and 153 are parent molecule isotopomers with ^13^C at natural abundance.

In Fig. 3, the ion ToF mass spectra for two different mixtures of enantiomeric vapours, Mix-RR (R-limonene/R-camphor) and Mix-RS (R-limonene/S-camphor), are shown after ionization with circular polarized 150 fs duration laser pulses with wavelength centred at 392.3 nm. The bandwidth of these fs laser pulses simultaneously encompasses two-photon resonant excitations to excited 3 s Rydberg states of either molecular species^24^,^30^, followed by absorption of a third photon taking each system above its ionization limit (Fig. 2a). Ion ToF-analysis produces similar mass spectra from both RS-(Fig. 3a) and RR-(Fig. 3b) mixtures. Only a small change in relative peak heights of the camphor versus limonene is observed between the Mix-RS and Mix-RR samples, due to slightly different experimental conditions, of no relevance here. The insets in Fig. 3 show the mass spectra in the region of 136–152 a.m.u., where the peaks corresponding to the parent ions of limonene (136 a.m.u.) and camphor (152 a.m.u.) can be clearly observed. From the width of the peaks in the mass spectra we can extract a mass resolution of m/Δm∼1,100 in this mass region. In this study we aimed for soft ionization with as low as possible fragmentation. The intensity and focus position of the femtosecond laser were adjusted such that the peaks due to fragmentation of the parent ion were only minor components in the mass spectra. As was reported before for ionization of pure camphor samples near 400 nm^24^, parent ion fragmentation can be easily induced by increasing the laser fluence although, noting the invariance of mass-selected photoelectron spectra (PES) of various ionic fragments, it was concluded that fragmentation was induced after photoionization of the neutral molecule. Hence, in principle, the laser fluence could be adjusted to obtain further structural identification from the fragmentation fingerprint^31^,^32^, and furthermore, the wavelength could be varied to achieve more selective ionization of preferred mixture components.

**Figure 4:**
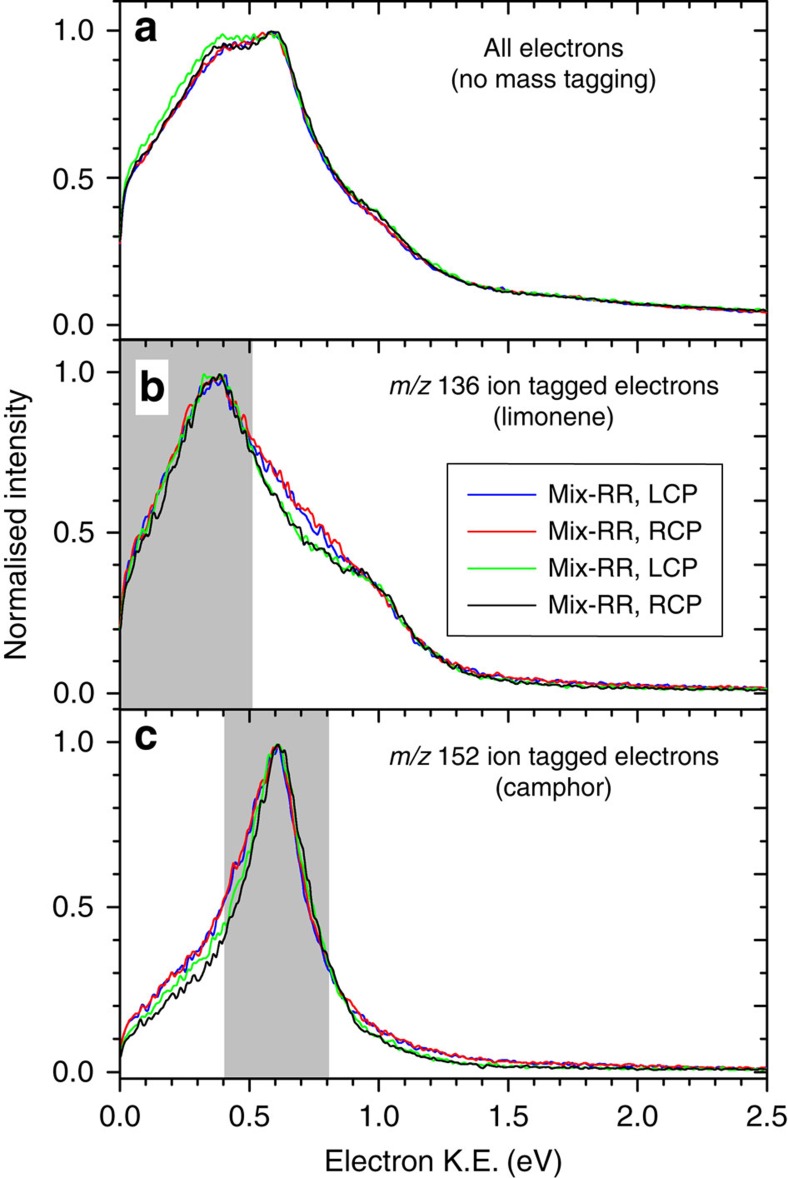
Photoelectron kinetic energy (K.E.) spectra. The mixtures Mix-RR and Mix-RS were irradiated with a femtosecond laser beam at 392 nm with either LCP or RCP. In panel (**a**) the kinetic energy of all detected electrons is shown; below that are shown the kinetic energy of coincident mass-tagged electrons at *m*/*z*=136 (limonene—panel (**b**)) and *m*/*z*=152 (camphor—panel (**c**)). The mass-tagged electrons in the shaded areas around the main peaks, (0.25±0.25) eV for limonene and (0.6±0.2) eV for camphor, were used to extract the three-dimensional angular distribution from which the PECD asymmetry was calculated, see Fig. 5.

In Fig. 4, we show the PES obtained after ionization at 392 nm with CPL. The electron kinetic energy distributions are extracted from their full three-dimensional momentum distributions recorded by position and time-of-arrival encoding of individual electrons at our imaging delay line detector^33^. Furthermore, the coincidence between ion and electron events allows each electron to be associated with formation of an ion of given mass. For each mixture PES are shown for ionization with both right and left circular polarized (RCP and LCP) light. In Fig. 4a the PES obtained from the arrival of all electrons without any correlation of the electron with the mass of the coincident ion are shown; in Fig. 4b the PES of electrons coincident with an ion mass 136 (limonene) are shown, and in Fig. 4c the spectra of electrons coincident with an ion mass 152 (camphor) are presented. As expected the PES for the mixtures Mix-RR and Mix-RS and the two light helicities are all the same for a given choice of electron mass-tagging. However, we do see clear differences between the mass-tagged electron spectra coincident with camphor or limonene parent ions within a single mixture. A prominent peak around 0.6 eV is observed in the camphor tagged PES. Using the ionization energy of camphor of 8.7 eV and a laser photon energy of 3.16 eV the electron peak at 0.6 eV is assigned to three-photon ionization of the highest occupied molecular orbital (MO)^24^, leaving only 0.18 eV of internal vibrational excitation in the camphor parent ion. The limonene tagged PES shows somewhat broader structures with a dominant peak around 0.3 eV. This peak is assigned to three-photon ionization of the second highest occupied MO^30^.

Multiphoton PECD measurements can be used to characterize the selected enantiomer by means of a simple asymmetry factor, defined for a given CPL as:^24^


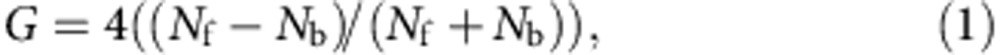


where *N*_f_ and *N*_b_ are, respectively, the number of electrons emitted into forward and backward hemispheres (that is, 0≤*θ*<π/2 and π/2≤*θ*<π relative to the light propagation direction) and are derived from the accumulated electron distribution data after filtering by coincident ion mass and the preferred energy range. In practice, measurements are made by alternating LCP and RCP light and the two counts, *N*_f_ and *N*_b_, obtained by combining the two polarization measurements in a manner that cancels any residual instrumental forward–backward asymmetry, as detailed below in Methods and section II of ref. 24).

**Figure 5:**
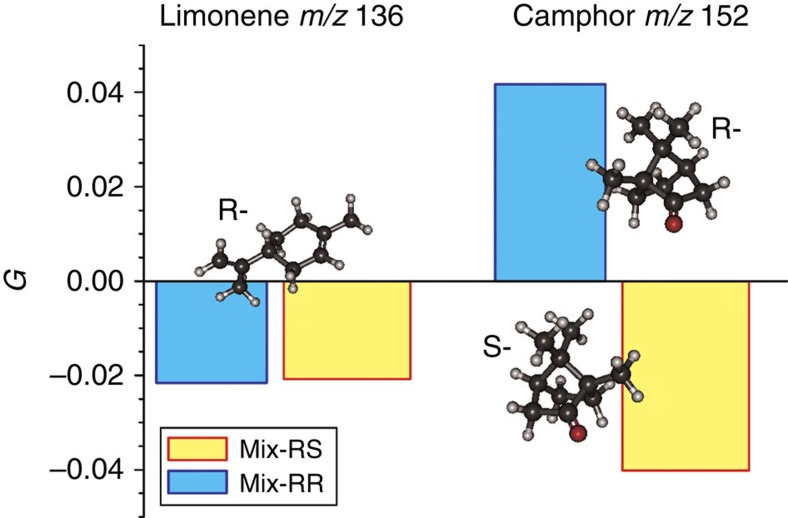
Mass-selected PECD asymmetries. Mass-selected PECD asymmetries, *G*, measured by multiphoton MS-PECD for two mixtures of enantiomerically pure limonene and camphor. Between Mix-RR (R-limonene/R-camphor) and Mix-RS (R-limonene/S-camphor) the camphor MS- PECD asymmetry flips sign, as anticipated for a change from R- to S-camphor, while for limonene the MS-PECD asymmetry remains the same. See also Table 1 for explicit values with uncertainties.

In Fig. 5 the mass-tagged PECD asymmetries for 392 nm excitation, determined across the respective PES peak ranges identified in Fig. 4, are plotted for Mix-RR and Mix-RS. It is observed that PECD is different from zero (absolute PECD values ∼2% and ∼4%), indicating that the compounds in this gaseous mixture at both the mass 136 and mass 152 are chiral. Furthermore, when changing from Mix-RR to Mix-RS the mass 136 (limonene) tagged PECD does not change (within experimental error, see Table 1), while for mass 152 (camphor) PECD reverses sign when changing the mixture. This latter sign reversal is anticipated and clearly correlates with the change of the camphor enantiomer in the mixture. Conversely, the absence of change in the mass 136 tagged PECD indicates that the enantiomer composition of limonene has not changed between the two mixtures. This is exactly in agreement with the composition of the two mixtures that we prepared in this experiment.

### Analysis for enantiomeric excess

As a final demonstration recordings were made with Mix-S[Rs], a combination of S-limonene and a 3:1 mix of R:S camphor vapours (50% enantiomeric excess (e.e.)). The results are compared with those for the previous two mixtures in Table 1. The S-limonene PECD retains the same magnitude but, as would be predicted, opposite sign to the previous R-limonene mixtures while the camphor PECD is reduced in magnitude, reflecting the reduction in enantiomeric purity.

For any component the observed PECD can be written as *G*=*f*_R_*G*_R_+*f*_S_*G*_S_=(2*f*_R_−1)*G*_R_, where *f*_*X*_ is the fractional abundance of enantiomer *X* and *G*_*X*_ its PECD asymmetry. But since *e*.*e*.=(*f*_R_*−f*_S_) × 100% it is straightforward to show that *e*.*e*.=*G*/*G*_R_ × 100%. We can obtain a value for camphor *G*_R_ under the conditions of these measurements, assuming 100% *e.e*. of our commercial R- and S- camphor products, from the mean magnitude measured for Mix-RR and Mix-RS, which from Table 1 gives *G*_R_= 0.041(2) where the uncertainty in the last digit is the sample s.d. Hence the measured camphor PECD for Mix-R[Rs] (Table 1) suggests the result *e.e.*=0.5±0.2, in excellent agreement with the prepared composition of this sample.

## Discussion

It is evident from the preceding results that the PECD asymmetry factor, *G* (equations (1) and (5)), evaluated simply from the electron emission yields in forward and backward directions (relative to the laser beam direction) provides a convenient, quantitative characterization of enantiomeric composition. These are identical to asymmetry factors obtained from the full analysis of the photoelectron angular distribution (see Methods section and Supplementary Table 1) allowing us to conclude that a full angular resolving electron detector is not a prerequisite for MS-PECD enantiomeric analysis; in principle a bi-detector, capable of discriminating just forward and backward electron scattering directions would be sufficient. Table 1 includes another comparison, this of results obtained with energy selection but without mass-tagging of the electrons. Even the partial overlapping of the PES regions assigned to camphor and limonene in Fig. 4 leads to a complete scrambling of the non-tagged PECD *G* values which no longer display an intuitively discernable pattern. Interpretation of such data therefore poses at best a considerably greater challenge and would require far greater *a priori* knowledge concerning the mixture under examination. The correlation of electron and ion data by coincidence detection methods therefore is capable of yielding a level of insight additional to either the electron- or ion mass-spectroscopy alone.

Many (chiral) molecules have two-photon absorptions accessible at single photon wavelengths ∼400 nm and ionization energies in a corresponding three-photon region around 9 eV. Combined with the large spectral bandwidth of an ultrafast laser pulse, the simultaneous ionization of multiple molecular components in a mixture can be expected to be relatively facile. Conversely, at longer wavelengths more selective ionization via selected chromophores becomes feasible. PECD itself provides a very rich signature of the ionized species, with asymmetries that vary depending on initial orbital, photon energy and molecular conformation^20^,^21^. Variation of the resonant laser ionization scheme would allow for effective exploitation of these properties, perhaps by employing more selective excitation through different resonant states (see Fig. 2) or by establishing conditions that optimize the magnitude of detectable PECD asymmetry—and hence further enhance sensitivity. (In this context it may be noted that despite the large excess of neon carrier gas (>99%) ionization of Ne is completely suppressed precisely because there is no accessible resonantly enhanced pathway with 392 nm photons, and a non-resonant mechanism would demand much higher laser intensity.) Furthermore, with picosecond lasers one can selectively excite individual vibrational bands within the electronically excited intermediate. This may provide opportunities for conformer specific resonant ionization^34^ and also open the door to possible rich vibronic phenomena in PECD capable of providing further structural insights^35^,^36^.

The precision of our e.e. determination is comparable to that achieved, with comparable data acquisition times, using a polarized microwave double resonance technique^12^,^13^,^37^ with detected phase providing enantiomer discrimination; for example, an e.e. for (-)-menthone was measured as 0.625±0.242^38^. Shorter acquisition times have been reported using a variation of this technique, permitting an e.e. of 0.025±0.005 to be measured with a 1,2-propanediol mixture with a known prepared 0.02 enantiomeric excess^39^. The asymmetry factors reported here are determined as a relatively simple function of two effective counters recording the number of electrons emitted into forward and backward hemispheres. These counts are assumed to be subject to Poisson statistics, which will determine the precision of the reported asymmetries, and especially the e.e. ratio. The reproducibility of repeated MS-PECD determinations, in both single and multi-component samples is, as shown in Table 1, very encouraging. Nevertheless, it is clear that an increase in data acquisition rate would provide benefits of reduced uncertainty associated with measurements and/or reduced measurement times with correspondingly improved convenience and sample consumption.

One obvious, and (in a limited sense) trivial to implement improvement would be an increase in laser repetition rate. A hundred-fold increase from the current 3 kHz, would yield a 10-fold improvement in statistical uncertainty, reducing that in the e.e. determination reported here to the level of a few %. Coincidence measurements with high (MHz) repetition rate sources are well established using synchrotron sources operating in their ‘few bunch' temporal modes; indeed the first electron–ion coincidence imaging PECD measurements were obtained in such conditions^40^. More recently, electron–ion coincidence experiments with a 400 kHz short pulse laser source have been reported^41^ and indeed suitable high rep-rate fibre lasers providing ps–fs pulse durations are now commercially available. An alternative, and not mutually exclusive, possibility would be a relaxation of the severe ionization rate limitation, here set at less than 0.08 electrons per laser shot, imposed to minimize false coincidences. The adoption of covariance mapping techniques to replace electron–ion coincidence detection^42^, may offer one route to accommodate significantly enhanced count rates without loss of the fundamental capabilities we have demonstrated. But this limitation can be eliminated completely if mass-tagged electron detection were not required for discriminating mixture components; for example if wavelength selective excitation/ionization techniques could instead be employed, or for e.e. determinations on single components. It is clear in such cases that, subject to detector dynamic range, ionization rates increased by three or more orders of magnitude could then be utilized. A combination of higher laser repetition rate and relaxation of the limiting ionization rate per laser shot can feasibly be expected to yield an order of magnitude improvement in both precision and acquisition times.

There is an alternative approach to enantioselective laser ionization mass spectrometry, LIMS-CD, that measures CD in the overall ion yield. To date, most reported experiments have utilized low-order (*n*+*n*′) resonance enhanced excitations by ns UV lasers^11^,^43^,^44^, although recently Weitzel and co-workers have demonstrated fs laser LIMS-CD, including non-resonant 800 nm excitation^10^. LIMS-CD and MS-PECD experiments with an ultrafast laser can thus share the potential capabilities of this ionization technique, as detailed above. Beyond that, while the (multiphoton) PECD effect develops in the pure electric-dipole approximation, LIMS-CD appears to depend on higher order multipole interactions, and in some way conflates CD contributions from various linear and nonlinear photon absorption steps. This can be evidenced by the observation of different CD from nascent parent ion and more photon hungry fragmentation channels^10^,^45^. In MS-PECD any post-ionization absorption should not contribute differently to the measured asymmetry.

Both LIMS-CD and MS-PECD require steps to reduce instrumental asymmetry. In LIMS-CD intensity differences in the LCP and RCP beams, and pulse-to-pulse fluctuations of the temporal and spatial distribution of the laser pulse energy are most effectively overcome for a single chiral component by concurrent measurement of an achiral reference which is thus required to have a similar absorption spectra and laser intensity dependence^10^,^44^,^46^—ideally for both parent and daughter ion channels. In some circumstances this might be quite demanding to arrange in a multi-component simultaneous measurement. In contrast, our MS-PECD data acquisition methodology (outlined in Methods, below) deliberately accumulates equal counts from the two polarizations such that when forming the dichroism as a difference between the two polarizations any residual, polarization-independent instrumental asymmetry in the angular distributions, for example, resulting from detector spatial inhomogeneities, gets fully cancelled. This precaution is trivially implemented for all species, but so doing actively precludes observation of any CD that may be present in the ionization yields.

A laboratory scale ultrafast laser imaging mass spectrometer capable of simultaneously detecting multiple enantiomeric species in gaseous mixtures without requiring prior separation techniques or reactions with complexing reagents, such as we have considered here, will open new analytical applications for the sensitive and selective detection of chiral molecules. As the measurements are performed on mass-selected species we can envisage extending the technique to molecular complexes like chiral dimers, or microsolvated chiral molecules^28^. Furthermore, the combination of MS-PECD with laser desorption of chiral molecules from site-selected regions of surface samples^15^, will extend the MS-PECD technique to sample chiral molecules directly from spatially selected regions on a two-dimensional surface^47^. A complementary approach using a table-top laser source of elliptically polarized VUV for single photon PECD measurements has also been recently described^48^.

Finally we note that theoretical modeling of single photon PECD is generally of sufficient quality to identify unambiguously absolute molecular configuration from the experimental observations^20^,^49^. The current theoretical modelling for multiphoton PECD is less developed^24^,^50^ although work such as that presented here should help stimulate further theoretical efforts to model and quantify multiphoton PECD.

## Methods

### Experimental setup

The experimental setup used in the present work has been described in detail before^33^ and is schematically shown in Fig. 1. The expanding gas mixture generates a continuous molecular beam in the source chamber which is doubly skimmed downstream of the nozzle as it passes through the buffer chamber into the imaging spectrometer chamber. In the imaging chamber the molecular beam intersects the laser beam at 90°. After ionization of a molecule, the electron and ion are detected in coincidence on two opposing time- and position-sensitive delay line detectors. In our experiment we operated velocity map imaging voltages for electron detection and optimized voltages for optimal mass resolution (and not necessarily optimal voltages for velocity map imaging of ions) for ion detection.

Camphor and limonene enantiomers—R and S forms—of high purity (>96%) were purchased from Sigma Aldrich. The liquid limonene samples were held in an external stainless steel reservoir maintained at room temperature. A stream of pure neon gas at 0.6 bar backing pressure sweeps the vapour of limonene through a stainless steel transfer line and through a further sample reservoir, containing camphor solids, also at room temperature. A continuous seeded molecular beam is created by expanding the gas mixture through a nozzle into the first vacuum chamber from where it is doubly skimmed before entering the ionization region. To prepare Mix-S[Rs] a 3:1 mixture of R:S camphor (*e.e*.=50%) was accurately weighed out. It was then ground together to ensure a homogeneous sample of the enantiomers before being placed in the reservoir.

A commercial femtosecond laser system manufactured by *Spectra Physics* was used. The laser had a pulse duration of about 150 fs with 3 kHz repetition rate, and the frequency doubled output at 392 nm was attenuated to provide pulse energies of ∼10–12 μJ (Mix-RR and Mix-S[Rs]) and 13–16 μJ (Mix-RS). It was loosely focused into the molecular beam with a lens of about 30 cm focal length. The corresponding intensity in the ionization volume is estimated to be 10^11^–10^12^ W cm^−2^. Further details are provided in Supplementary Figs 1–4 and Supplementary Methods.

### Data acquisition and analysis

For each mixture data were accumulated for ∼2 × 10^8^ laser shots, during which the circular polarization (LCP/RCP) was switched every 500 s in order to reduce the effect of any experimental drift. The average number of electron events per laser shot, independent of coincident ion was limited to about 0.08. This count rate ensures that the probability for false coincidences is less than 0.3% assuming Poisson statistics.

The photoelectron angular distribution, *I*_*p*_*(θ),* arising from an *n*-photon ionization of a chiral molecule with circular polarized light is given by^24^


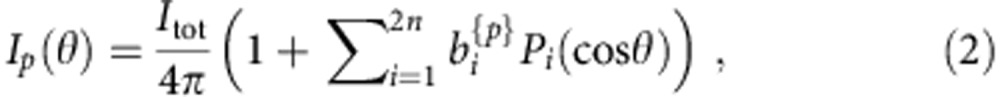


where *p* is the polarization (*p*=+1 and *p*=−1 correspond to LCP and RCP radiation, respectively). Here, *θ* is the angle between the electron momentum and the propagation direction of the laser beam, *P*_*i*_ (cos*θ*) are Legendre polynomials and 
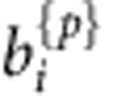
are the corresponding coefficients. Because the odd 
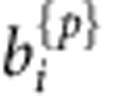
coefficients are anti-symmetric with respect to circular polarization switching (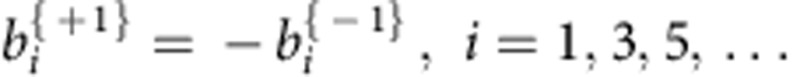), whereas the even coefficients are unaltered^21^,^22^,^24^ the chiral (odd) coefficients can be isolated by forming the dichroism or difference angular distribution:


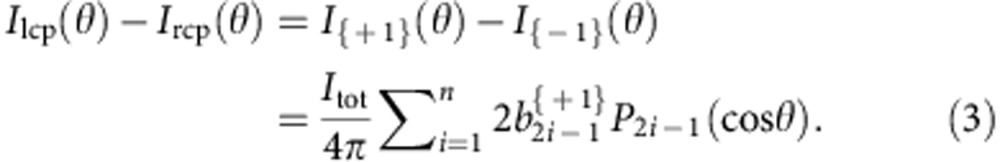


Forward–backward asymmetry arises from these odd polynomials in cos(*θ*). A useful measure to quantify the enantiomeric sensitivity of multiphoton PECD in a single asymmetry parameter has been suggested as


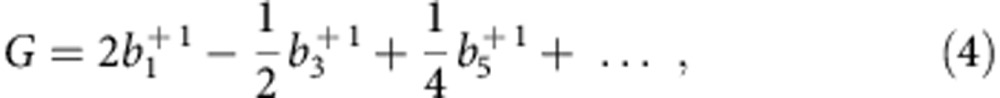


(truncated as shown for a three-photon process) and this can be trivially evaluated from considering, for each polarization *p*, the numbers of electrons emitted in the forward and backward hemispheres (respectively *N*_*p,*f_ and *N*_*p,*b_ ):^21^,^22^,^24^


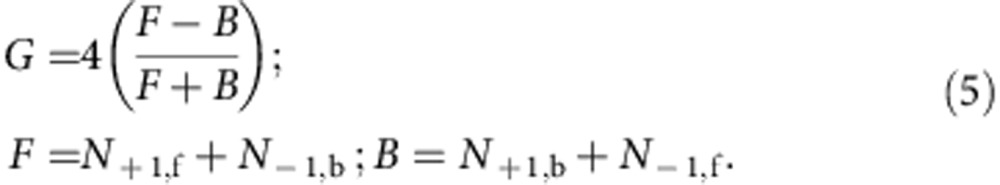


This form of combining polarization measurements has the experimental advantage of cancelling any polarization-independent residual instrumental asymmetry that might result, for example, from detector gain inhomogeneity. To ensure optimum cancellation it is arranged that we achieve the same total count for each polarization such that (*N*_+1,f_+*N*_+1,b_)=(*N*_−1,f_+*N*_−1,b_). Assuming Poisson counting statistics there is an uncertainty 
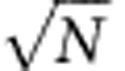
 in each count and so the error in *G* is obtained by standard error propagation.

Mass-tagged and photoelectron-energy selected coincidence events were filtered from the data for the various polarization settings, and PECD asymmetry factors calculated using equation (5) are presented in Table 1, with errors estimated by error propagation assuming Poisson counting statistics.

Further experimental details are provided in Supplementary Methods and we also compare *G* factors derived from the full electron angular distribution analysis (equation (4)) with those from application of equation (5) in Supplementary Table 1.

## Additional information

**How to cite this article:** Fanood, M. M. R. *et al.* Enantiomer-specific analysis of multi-component mixtures by correlated electron imaging–ion mass spectrometry. *Nat. Commun.* 6:7511 doi: 10.1038/ncomms8511 (2015).

## Supplementary Material

Supplementary Information Supplementary Figures 1-4, Supplementary Table 1, Supplementary Methods and Supplementary References

## Figures and Tables

**Table 1:** MS-PECD asymmetry factor *G* * measured for the various mixtures and pure samples.

	**Mass-tagged data**		**Non-mass-tagged data**	
**Component**	**Limonene**	**Camphor**	**All ions**	**All ions**
**Ion Mass (m/z)**	**136**	**152**		
**Electron Energy Range (eV)**	**0.0–0.5**	**0.4–0.8**	**0.0–0.5**	**0.4–0.8**
Mix-RR	−0.022(9)	0.042(11)	−0.003(4)	0.023(5)
Mix-RS	−0.023(9)	−0.039(12)	−0.019(4)	−0.015(4)
Mix-S[Rs]	0.024(7)	0.022(9)	0.017(4)	0.020(4)
				
Pure R-limonene	−0.019(11)			
Pure S-limonene	0.020(11)			
				
Mean **|*****G*****|**	0.022(2)†	0.041(2)‡		

^*^G value derived by forward/backward differences in coincident electron counts, see equation 5. Quoted uncertainties (in brackets of last digits) are estimated assuming Poisson counting statistics and using standard error propagation. The filtered electron energy ranges are those shown shaded in Fig. 4b and Fig. 4c.

^†^Mean absolute value of MS-PECD *G* for limonene obtained from the five values above. The sign corresponds to the value for S-limonene and ionization with LCP light. The s.d. of the sample (in brackets of last digits) is shown.

^‡^Mean absolute value of MS-PECD *G* for camphor obtained from the two values above and excluding the value of the enantiomerically mixed camphor in Mix-S[Rs]. The sign corresponds to the value for R-camphor and ionization with LCP light. The s.d. of the sample (in brackets of last digits) is shown.

## References

[b1] Brenna E., Fuganti C. & Serra S. Enantioselective perception of chiral odorants. Tetrahedron-Asymmetry 14, 1–42 (2003).

[b2] Song W., Staudt M., Bourgeois I. & Williams J. Laboratory and field measurements of enantiomeric monoterpene emissions as a function of chemotype, light and temperature. Biogeosciences 11, 1435–1447 (2014).

[b3] Eerdekens G. *et al.* VOC measurements within a boreal forest during spring 2005: on the occurrence of elevated monoterpene concentrations during night time intense particle concentration events. Atmos. Chem. Phys. 9, 8331–8350 (2009).

[b4] Biasioli F., Yeretzian C., Mark T. D., Dewulf J. & Van Langenhove H. Direct-injection mass spectrometry adds the time dimension to (B)VOC analysis. Trac-Trends Anal. Chem 30, 1003–1017 (2011).

[b5] Deleris I. *et al.* Comparison of direct mass spectrometry methods for the on-line analysis of volatile compounds in foods. J. Mass Spectrom. 48, 594–607 (2013). 23674284 10.1002/jms.3199

[b6] Tao W. A., Gozzo F. C. & Cooks R. G. Mass spectrometric quantitation of chiral drugs by the kinetic method. Anal. Chem. 73, 1692–1698 (2001). 11338581 10.1021/ac001150v

[b7] Wu L. & Vogt F. G. A review of recent advances in mass spectrometric methods for gas-phase chiral analysis of pharmaceutical and biological compounds. J. Pharm. Biomed. Anal. 69, 133–147 (2012). 22579598 10.1016/j.jpba.2012.04.022

[b8] Berova N., Nakanishi K. & Woody R. Circular dichroism principles and applications 2nd edn Wiley-VCH (2000).

[b9] Berova N., Polavarapu P., Nakanishi K. & Woody R. Comprehensive chiroptical spectroscopy John Wiley & Sons (2012).

[b10] Horsch P., Urbasch G. & Weitzel K. M. Analysis of chirality by femtosecond laser ionization mass spectrometry. Chirality 24, 684–690 (2012). 22544648 10.1002/chir.22037

[b11] Boesl U., Bornschlegl A., Loge C. & Titze K. Resonance-enhanced multiphoton ionization with circularly polarized light: chiral carbonyls. Anal. Bioanal. Chem. 405, 6913–6924 (2013). 23430178 10.1007/s00216-012-6666-3

[b12] Patterson D., Schnell M. & Doyle J. M. Enantiomer-specific detection of chiral molecules via microwave spectroscopy. Nature 497, 475 (2013). 23698447 10.1038/nature12150

[b13] Shubert V. A., Schmitz D., Patterson D., Doyle J. M. & Schnell M. Identifying enantiomers in mixtures of chiral molecules with broadband microwave spectroscopy. Angew. Chem. Int. Ed 53, 1152–1155 (2014). 10.1002/anie.201306271 24311230

[b14] Mukhopadhyay P., Zuber G., Wipf P. & Beratan D. N. Contribution of a solute's chiral solvent imprint to optical rotation. Angew. Chem. Int. Ed 46, 6450–6452 (2007). 10.1002/anie.200702273 17645276

[b15] de Vries M. S. & Hobza P. Gas-phase spectroscopy of biomolecular building blocks, theory and experiment. Annu. Rev. Phys. Chem. 58, 585–612 (2007). 17291183 10.1146/annurev.physchem.57.032905.104722

[b16] Zehnacker A. & Suhm M. A. Chirality recognition between neutral molecules in the gas phase. Angew. Chem. Int. Ed 47, 6970–6992 (2008). 10.1002/anie.200800957 18696527

[b17] Zehnacker A. Chiral recognition in the gas phase Taylor & Francis (2010).

[b18] Bodi A., Hemberger P., Osborn D. L. & Sztaray B. Mass-resolved isomer-selective chemical analysis with imaging photoelectron photoion coincidence spectroscopy. J. Phys. Chem. Lett. 4, 2948–2952 (2013).

[b19] Ritchie B. Theory of the angular distribution of photoelectrons ejected from optically active molecules and molecular negative ions. Phys. Rev. A 13, 1411–1415 (1976).

[b20] Powis I. in Adv. Chem. Phys. ed. Light J. C. Vol. 138, 267–329Wiley (2008).

[b21] Janssen M. H. M. & Powis I. Detecting chirality in molecules by imaging photoelectron circular dichroism. Phys. Chem. Chem. Phys. 16, 856–871 (2014). 24305955 10.1039/c3cp53741b

[b22] Ram N. B., Lehmann C. S. & Janssen M. H. M. Probing chirality with a femtosecond reaction microscope. EPJ Web Conf. 41, 02029 (2013).

[b23] Lux C. *et al.* Circular dichroism in the photoelectron angular distributions of camphor and fenchone from multiphoton ionization with femtosecond laser pulses. Angew. Chem. Int. Ed. 51, 5001–5005 (2012). 10.1002/anie.201109035 22351439

[b24] Lehmann C. S., Ram N. B., Powis I. & Janssen M. H. M. Imaging photoelectron circular dichroism of chiral molecules by femtosecond multiphoton coincidence detection. J. Chem. Phys. 139, 234307 (2013). 24359367 10.1063/1.4844295

[b25] Rafiee Fanood M. M., Powis I. & Janssen M. H. M. Chiral asymmetry in the multiphoton ionization of methyloxirane using femtosecond electron-ion coincidence imaging. J. Phys. Chem. A 118, 11541–11546 (2014). 25402546 10.1021/jp5113125

[b26] Lux C., Wollenhaupt M., Sarpe C. & Baumert T. Photoelectron circular dichroism of bicyclic ketones from multiphoton ionization with femtosecond laser pulses. Chemphyschem. 16, 115–137 (2015). 25492564 10.1002/cphc.201402643

[b27] Rafiee Fanood M. M., Janssen M. H. M. & Powis I. Enantioselective femtosecond laser photoionization spectrometry of limonene using photoelectron circular dichroism. Phys. Chem. Chem. Phys. 17, 8614–8617 (2015). 25744283 10.1039/c5cp00583c

[b28] Powis I. *et al.* A photoionization investigation of small, homochiral clusters of glycidol using circularly polarized radiation and velocity map electron-ion coincidence imaging. Phys. Chem. Chem. Phys. 16, 467–476 (2014). 24077129 10.1039/c3cp53248h

[b29] Vredenborg A., Lehmann C. S., Irimia D., Roeterdink W. G. & Janssen M. H. M. The reaction microscope: imaging and pulse shaping control in photodynamics. Chemphyschem. 12, 1459–1473 (2011). 21506237 10.1002/cphc.201100107

[b30] Smialek M. A. *et al.* Limonene: electronic state spectroscopy by high-resolution vacuum ultraviolet photoabsorption, electron scattering, He(I) photoelectron spectroscopy and ab initio calculations. Phys. Chem. Chem. Phys. 14, 2056–2064 (2012). 22231475 10.1039/c2cp22847e

[b31] Lozovoy V. V. *et al.* Control of molecular fragmentation using shaped femtosecond pulses. J. Phys. Chem. A 112, 3789–3812 (2008). 18433144 10.1021/jp071691p

[b32] Duffy M. J. *et al.* Fragmentation of neutral amino acids and small peptides by intense, femtosecond laser pulses. J. Am. Soc. Mass Spectrom. 24, 1366–1375 (2013). 23817831 10.1007/s13361-013-0653-6

[b33] Vredenborg A., Roeterdink W. G. & Janssen M. H. M. A photoelectron-photoion coincidence imaging apparatus for femtosecond time-resolved molecular dynamics with electron time-of-flight resolution of σ=18ps and energy resolution ΔE/E=3.5%. Rev. Sci. Inst 79, 063108 (2008). 10.1063/1.2949142 18601398

[b34] Karaminkov R., Chervenkov S., Delchev V. & Neusser H. J. High-resolution mass-selective uv spectroscopy of pseudoephedrine: evidence for conformer-specific fragmentation. J. Phys. Chem. A 115, 9704–9713 (2011). 21755930 10.1021/jp201399s

[b35] Powis I. The influence of vibrational parity in chiral photoionization dynamics. J. Chem. Phys. 140, 111103 (2014). 24655165 10.1063/1.4869204

[b36] Garcia G. A., Nahon L., Daly S. & Powis I. Vibrationally induced inversion of photoelectron forward-backward asymmetry in chiral molecule photoionization by circularly polarized light. Nat. Commun 4, 2132 (2013). 23828557 10.1038/ncomms3132 PMC3715848

[b37] Patterson D. & Schnell M. New studies on molecular chirality in the gas phase: enantiomer differentiation and determination of enantiomeric excess. Phys. Chem. Chem. Phys. 16, 11114–11123 (2014). 24801943 10.1039/c4cp00417e

[b38] Shubert V. A., Schmitz D. & Schnell M. Enantiomer-sensitive spectroscopy and mixture analysis of chiral molecules containing two stereogenic centers—microwave three-wave mixing of menthone. J. Mol. Spectrosc 300, 31–36 (2014).

[b39] Patterson D. & Doyle J. M. Sensitive chiral analysis via microwave three-wave mixing. Phys. Rev. Lett. 111, 023008 (2013). 23889397 10.1103/PhysRevLett.111.023008

[b40] Garcia G. A. *et al.* Circular dichroism in the photoelectron angular distribution from randomly oriented enantiomers of camphor. J. Chem. Phys. 119, 8781–8784 (2003).

[b41] Furch F. J. *et al.* Carrier-envelope phase stable few-cycle pulses at 400 kHz for electron-ion coincidence experiments. Opt. Express 21, 22671–22682 (2013). 24104154 10.1364/OE.21.022671

[b42] Mikosch J. & Patchkovskii S. Coincidence and covariance data acquisition in photoelectron and -ion spectroscopy. II. Analysis and applications. J. Mod. Opt 60, 1439–1451 (2013).

[b43] Li R., Sullivan R., Al-Basheer W., Pagni R. M. & Compton R. N. Linear and nonlinear circular dichroism of R-(+)-3-methylcyclopentanone. J. Chem. Phys. 125, 144304 (2006). 17042587 10.1063/1.2338519

[b44] Loge C. & Boesl U. Multiphoton ionization and circular dichroism: new experimental approach and application to natural products. Chemphyschem. 12, 1940–1947 (2011). 21506235 10.1002/cphc.201100035

[b45] Loge C. & Boesl U. Laser mass spectrometry with circularly polarized light: circular dichroism of molecular ions. Chemphyschem. 13, 4218–4223 (2012). 23090920 10.1002/cphc.201200614

[b46] Titze K., Zollitsch T., Heiz U. & Boesl U. Laser mass spectrometry with circularly polarized light: circular dichroism of cold molecules in a supersonic gas beam. Chemphyschem. 15, 2762–2767 (2014). 25044352 10.1002/cphc.201402270

[b47] Jungmann J. H. & Heeren R. M. A. Emerging technologies in mass spectrometry imaging. J. Proteomics 75, 5077–5092 (2012). 22469858 10.1016/j.jprot.2012.03.022

[b48] Ferré A. *et al.* A table-top ultrashort light source in the extreme-ultraviolet for circular dichroism experiments. Nature Photon. 9, 93–98 (2015).

[b49] Powis I. in Comprehensive chiroptical spectroscopy eds Berova N., Polavarapu P., Nakanishi K., Woody R. Vol. 1, pp 407–431John Wiley & Sons (2012).

[b50] Dreissigacker I. & Lein M. Photoelectron circular dichroism of chiral molecules studied with a continuum-state-corrected strong-field approximation. Phys. Rev. A 89, 053406 (2014).

