# Supplementary material for: Enantiomer-specific analysis of multi-component mixtures by correlated electron imaging–ion mass spectrometry
Source: Nat Commun. 2015 Jun 24;6:7511. doi: 10.1038/ncomms8511 (PMC4491818; doi:10.1038/ncomms8511)
Supplement: Supplementary Information — Supplementary Figures 1-4, Supplementary Table 1, Supplementary Methods and Supplementary References [file ncomms8511-s1.pdf]

Supplementary Figures

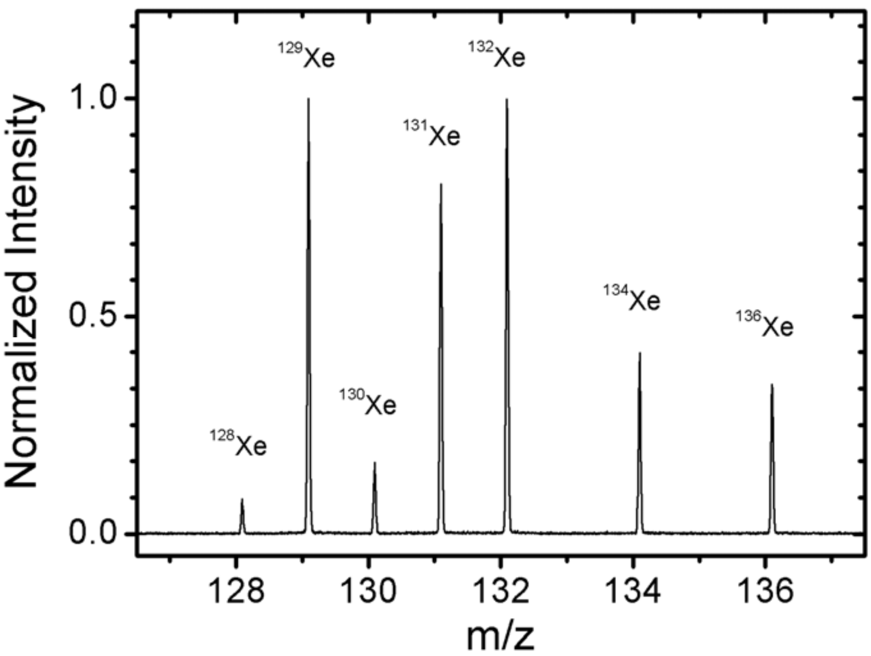

Supplementary Fig. 1. Typical mass spectrum as obtained on a seeded beam of 5% xenon in neon. The mass spectrum is measured under similar experimental conditions regarding laser and imaging spectrometer settings as the mixtures of limonene and camphor. All the isotopes of xenon can be clearly observed with good mass separation.

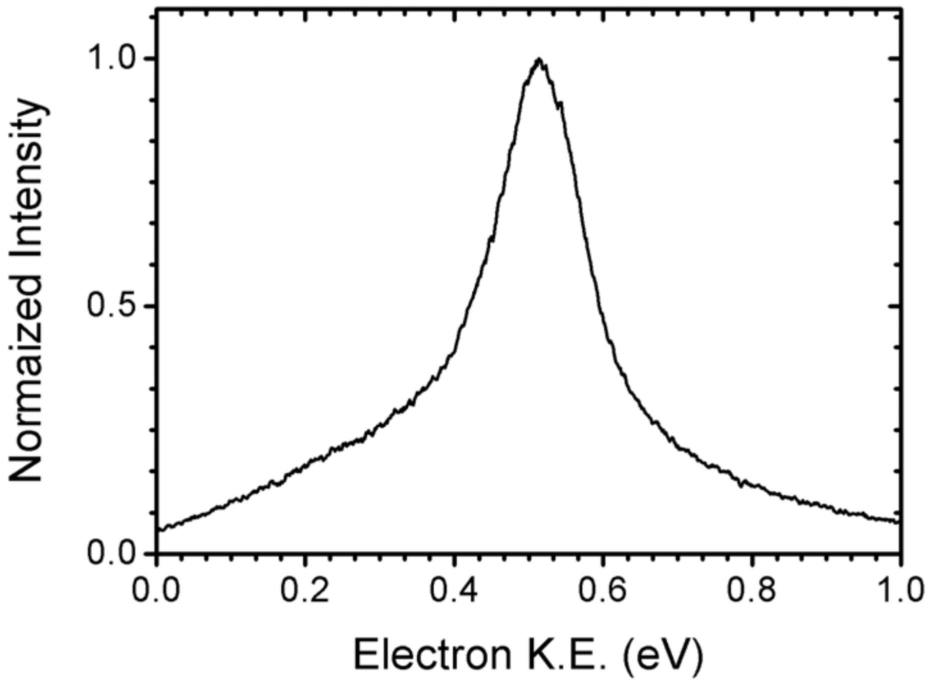

Supplementary Fig. 2. Typical photoelectron spectrum as measured in coincidence with Xe^{+} ions. With the ionization energy of xenon (IP = 12.12984 eV) and the measured wavelength of the femtosecond laser it can be concluded that the electrons result from 4 photon ionization. The electron kinetic energy can then be calibrated using energy conservation: 4*photon energy – IP = photoelectron energy.

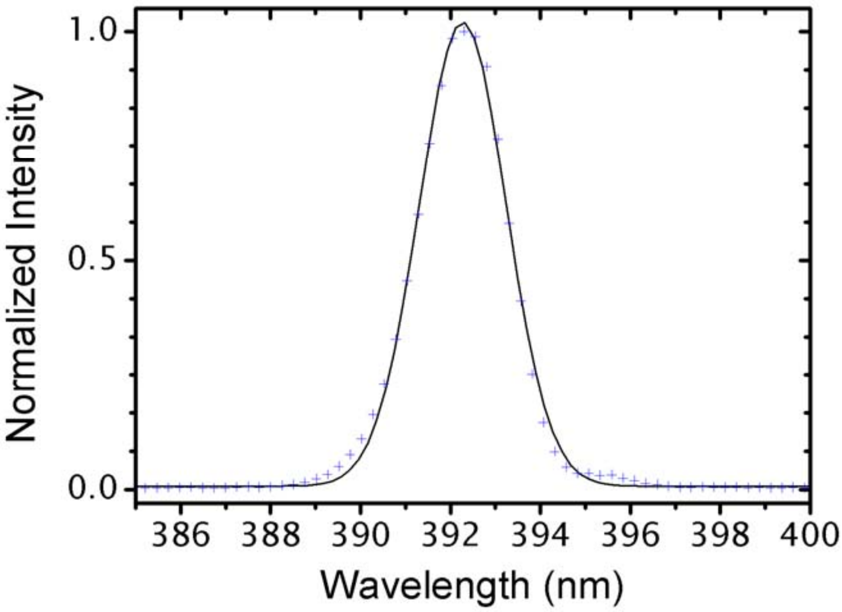

Supplementary Fig. 3. Typical spectrum of the frequency doubled femtosecond laser pulse with a central wavelength of 392.3 nm and a Full-Width-Half-Maximum of 2.3 nm.

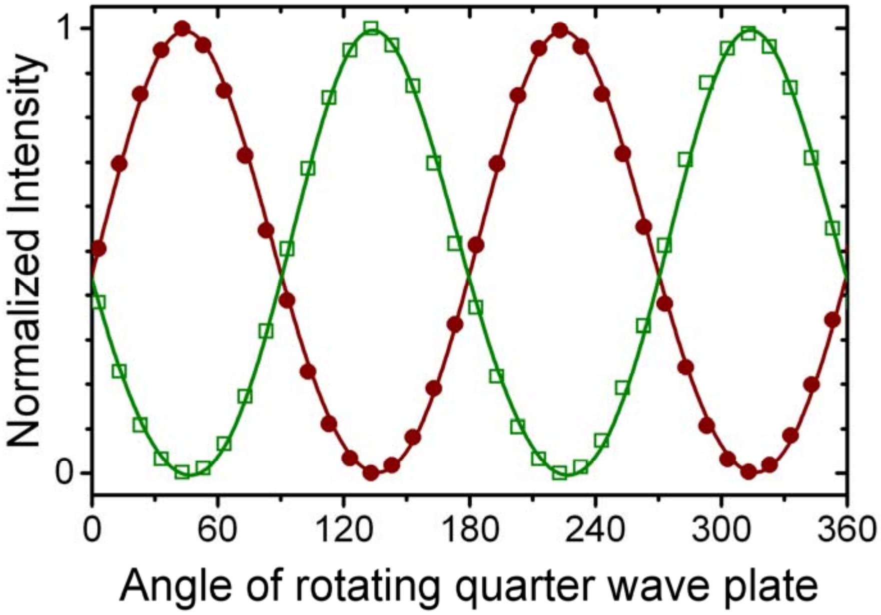

Supplementary Fig. 4. Measured graph for the analysis of the Stokes parameters of the circularly polarized laser pulses near 392 nm using the rotating quarter wave plate method[1]. The green data (data points are green squares, best fit is green line) was measured for LCP, the dark red data (data points are solid dots, best fit is red line) for RCP. The Stokes parameters obtained from the best fit lines are given in the text.

Supplementary Tables

Supplementary Table 1. A comparison of MS-PECD *G* values, obtained by two alternative analyses (see main text) for the various multi-component mixtures.

	Fwd/Bkwd Analysis[a]		LP Analysis[b]	
Component	limonene	camphor	limonene	camphor
Ion Mass (m/z)	136	152	136	152
Electron Energy Range (eV)	0.0–0.5	0.4–0.8	0.0–0.5	0.4–0.8
Mix-RR	-0.022(9)	0.042(11)	-0.025(11)	0.042(8)
Mix-RS	-0.023(9)	-0.039(12)	-0.022(11)	-0.043(16)
Mix-S[Rs]	0.024(7)	0.022(9)	0.025(7)	0.025(6)

[a] *G* value derived by Forward/Backward differences in coincident electron counts, see Eq. 5. Quoted uncertainties (in brackets of last digits) are estimated assuming Poisson counting statistics and using standard error propagation.

[b] *G* value derived from fitted electron angular distribution Legendre polynomial coefficients, see Eq. 4. Quoted uncertainties (in brackets of last digits) are derived from the errors in the fitted Legendre polynomial coefficients and using standard error propagation.

Supplementary Methods

Electron-ion coincidence imaging apparatus

The experimental setup used in the present work, shown schematically in Figure 1, has been described in detail before.[2] In brief, the coincidence imaging apparatus consists of three UHV chambers, the source chamber, a buffer chamber and the imaging chamber. A continuous molecular beam is generated by expansion through a 150 μm diameter conical nozzle into the source chamber and is doubly skimmed downstream of the nozzle by a 500 μm diameter skimmer followed by a 200 μm diameter skimmer as it passes through the buffer chamber into the imaging spectrometer chamber. The overall distance between nozzle and interaction region is about 45 cm. In the imaging chamber the molecular beam intersects the laser beam at 90°. The electron and ion resulting from a molecule's ionization are detected in coincidence on two opposing time- and position-sensitive delay line detectors. High voltage switches are used to change the magnitude and polarity of the high voltages on the ion lenses. The switches are operated at the repetition rate of the laser system (3 kHz). Both electron and ion time-of-flight (ToF) tubes are shielded by a 1mm thick μ-metal tube. In our experiment we operated velocity map imaging (VMI) voltages for electron detection and tuned voltages for optimal mass resolution (and not necessarily optimal voltages for VMI of ions) for ion detection. The typical voltages on the particle lenses (repeller (R), extractor (E) and extra lens (L), see Figure 2 in ref.[2]) that were operated for detecting electrons are R_e= -520 V, E_e= -385 V, L_e=-275 V and for detecting ions are R_i=2000 V, E_i=1460 V, L_i=0 V.

Energy calibration of the electron detector

The three-dimensional (3D) electron velocity distribution is obtained directly from the position encoding delay-line detector and Micro-Channel-Plate arrival time pickup.[2] Unlike the more conventional arrangement using CCD camera imaging of the 2D projection image of (non-coincident) photoelectrons, no inversion routine (like Abel transformation or pBasex deconvolution[3]) is needed to recover data. The energy scale and the origin of the 3D electron velocity distribution were carefully calibrated on multiphoton ionization of a seeded beam of 5% Xe in Ne. In Supplementary Figure 1 we show a typical mass spectrum of Xe, showing all the isotopes with clear mass resolution. In Supplementary Figure 2 a typical photoelectron spectrum as measured for Xe is shown. These calibration images on Xe photoionization were taken under the same conditions, using the same position and polarization settings of the laser beam, within the same experimental runs. This allows for a careful analysis of the calibration of the photoelectron images.

Laser system, characterization of spectrum and polarization of pulses

In the present experiments a commercial femtosecond laser system manufactured by Spectra Physics was used. It consists of a Titanium:Sapphire oscillator (Mai-Tai) that seeds the chirped regenerative amplifier (Spitfire-Ace). The output of the amplifier was optimized to deliver pulses centered at 784.6 nm wavelength with >5 W power at 3 kHz repetition rate and 120-150 fs pulse duration. The fundamental beam is subsequently frequency doubled in a BBO crystal. The spectral width and centre wavelength are continuously monitored with a fiber-based spectrometer (Ocean Optics USB 4000). In Supplementary Figure 3 a typical spectrum of the frequency doubled femtosecond laser pulse near 392.3 nm is shown.

A broadband quarter wave plate (B-Halle) was used to generate circular polarized light. The polarization of the light was characterized in terms of the Stokes vector by the rotating quarter wave plate method.[1] The measured intensity curves for both left- and right circular polarized light for the pulses at 392 nm are shown in Supplementary Figure 4 and the Stokes vectors obtained from the curves are: RCP: **S** = (1,-0.01,0.03,0.99) and LCP: **S** = (1, -0.11, 0.08, -0.99). Hence, we achieve nearly perfect circular polarization of the laser light source ($S_3 \approx \pm 1$).

Sample composition and consumption

The samples of limonene/camphor, held in room temperature reservoirs, are co-expanded with 0.6 bar Ne behind the nozzle into the spectrometer. Taking the room temperature sample vapour pressures to be 2 mbar (limonene) and 0.5 mbar (camphor)[4] we can estimate the respective composition in the molecular beam, assuming seeding fractions correspond to the relative partial pressures, to be 0.33% and 0.08%, respectively. An estimate of the sample consumption can be made using the pressure and pump speed in the source chamber. Given the observed pressure increase in the source chamber to be about $2*10^{-4}$ mbar when the beam is on, we can derive an estimate for the partial pressure of limonene as $(2\times10^{-3}/0.6)\times2\times10^{-4}$ mbar = 6.7×10^{-7} mbar. With an effective pump speed of about 1000 liter per sec in the source chamber the pumped limonene sample volume is $1000\times6.7\times10^{-7}$ mbar liter per sec = 6.7×10^{-7} bar liter per sec ~ 2.7×10^{-8} mol per sec of limonene sample. In the present experiment we measured about 10^8 laser shots per polarization which corresponds to 7×10^4 sec for a complete measurement made with the current laser rep rate of 3 kHz. From this we estimate that we used about 1.9 mmol of limonene. Consumption of camphor, which has a vapour pressure of about 25% relative to limonene, will therefore be about 0.5 mmol.

Measurement procedure and data analysis

The measurements on each mixture were performed switching between LCP and RCP polarization at 500 sec intervals. After careful checks that, among other things, the centre of the electron data are the same (by checking the xenon data files taken during the

measurements), all files with the same polarization were combined together at the end of the measurement for data analysis. The coincident data on electrons and ions provide direct time and position information for each event that can be converted directly (after calibration of the electron and ion spectrometers with Xe) to ion mass and full three-dimensional momenta (p_x, p_y, p_z) of the electron. The latter is in turn reduced further to an emission direction into either the forward or backward direction (relative to the laser beam).

Under our present experimental conditions of laser fluence, sample density and detector efficiencies, we detect about 0.08 electrons per laser shot, and obtain about 0.012 detected (e,ion) coincidence events per laser shot. Selection of ion mass and electron energy range filters a total of about 50000–80000 mass-tagged, energy selected (e,ion) events out of the total set of coincidence events per LCP or RCP polarization measurement per mixture.

Although equal recording time is devoted to the two polarizations, small intensity differences etc. mean that there is a need to adjust the two data sets to have the same total event count after which the PECD asymmetry, *G*, is calculated using Eq. 5. Because of this adjustment we are effectively insensitive to any differences of overall detection efficiency between LCP and RCP in this multiphoton excitation scheme.

The analysis (see main text) using the forward/backward differences in events is compared with the alternative analysis of fitting the full angular distribution of electron scattering by a Legendre polynomial series up to rank six in the Supplementary Table 1. As expected these two alternative analysis methods to extract MS-PECD *G*-values agree very well. Hence the forward/backward analysis presented in the main paper and Figure 5 can be judged as a representative method to report MS-PECD *G*-values.

Supplementary References

[1] Schaefer, B., Collett, E., Smyth, R., Barrett, D., Fraher, B., Measuring the Stokes polarization parameters. *Am. J. Phys.* **75**, 163-168 (2007).

[2] Vredenborg, A., Roeterdink, W. G., and Janssen, M. H. M., A photoelectron-photoion coincidence imaging apparatus for femtosecond time-resolved molecular dynamics with electron time-of-flight resolution of σ=18 ps and energy resolution Δ E/E=3.5%. *Rev. Sci. Inst.* **79**, 063108 (2008).

[3] Garcia, G. A., Nahon, L., and Powis, I., Two-dimensional charged particle image inversion using a polar basis function expansion. *Rev. Sci. Inst.* **75**, 4989-4996 (2004).

[4] Yaws, Carl L., *Yaws' Critical Property Data for Chemical Engineers and Chemists*. (Knovel, Norwich, N.Y., 2014).
